# Supplementary material for: The FAM114A proteins are adaptors for the recycling of Golgi enzymes
Source: J Cell Sci. 2024 Sep 6;137(17):jcs262160. doi: 10.1242/jcs.262160 (PMC11441981; doi:10.1242/jcs.262160)
Supplement: Supplementary information [file joces-137-262160-s1.pdf]

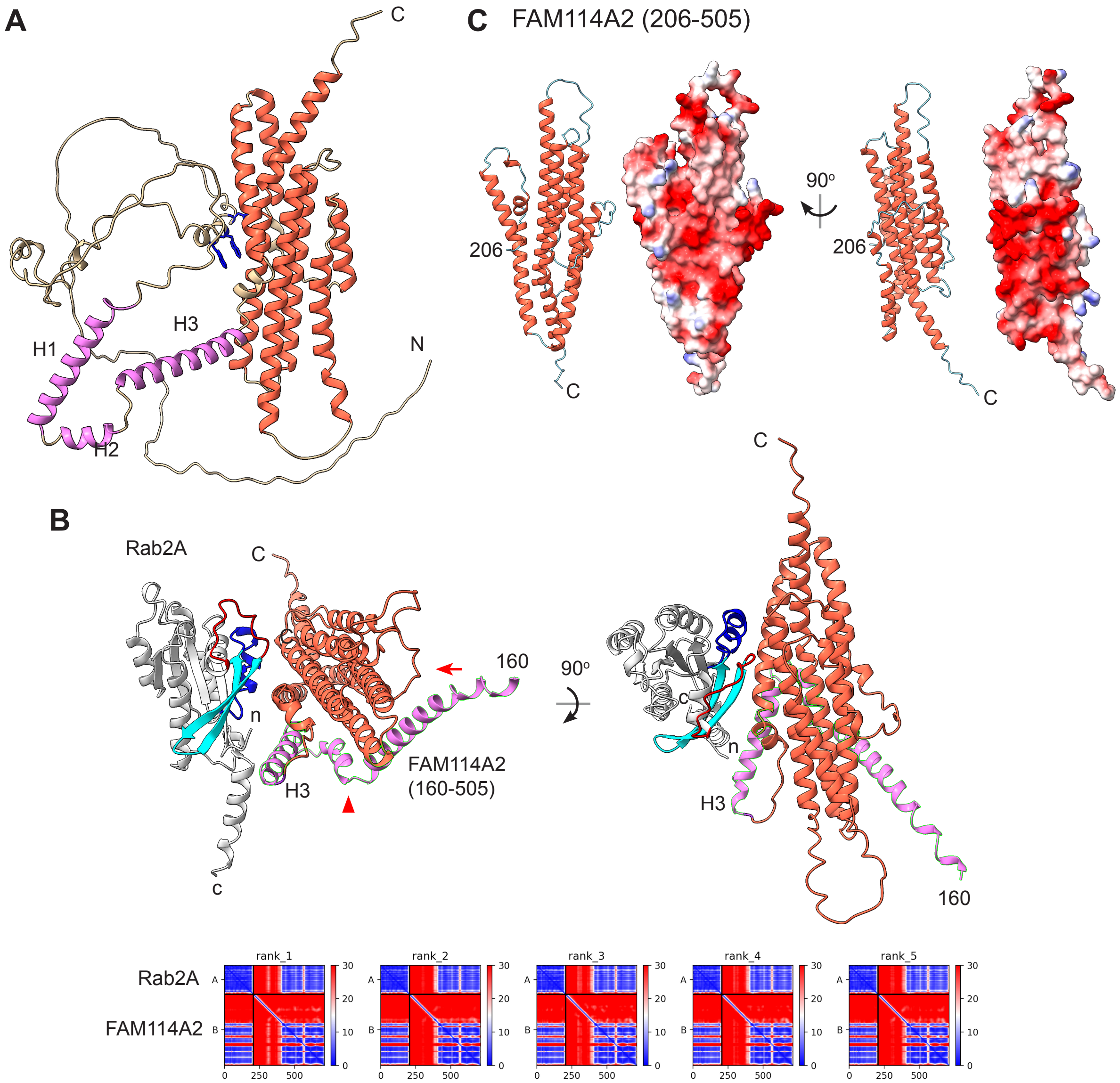

Fig. S1. Predicted structures for FAM114A2.

(A) AlphaFold2 prediction for the structure of human FAM11A2. From three recycles using mmseqs2_uniref as MSA mode and default settings in ColabFold v1.5.5. The C-terminal helical bundle in brick-red and preceding three helices in violet. The three tryptophans in the WG motif region are shown in blue.

(B) AlphaFold prediction for the structure of human FAM11A2 in complex with Rab2A. From three recycles using mmseqs2_uniref as MSA mode and default settings in ColabFold, along with the PAE plot of all five models (lower scores are higher confidence and in blue). The interaction is predicted with high confidence (ipTM=0.893). For FAM114A2 the unstructured region (1-159) is omitted for clarity and colouring is as in (A). Rab2A is in grey with Switch-1 (red), interswitch (red) and Switch-2 (blue) indicated. As expected for a Rab:effector interaction, the Switch and interswitch regions make contact with the FAM114A2 helical bundle. Helix 3 is predicted to also pack against Rab2A, but this has not been verified experimentally. The predicted Rab2A binding site is away from the two negative faces shown in (C), as indicated by the triangle (right panel of (C)) and arrowhead (right panel).

(C) Electrostatic potential plot of the surface of the C-terminal helical bundle – red is negative, blue is positive. Two highly negative surfaces are present.

Journal of Cell Science • Supplementary information

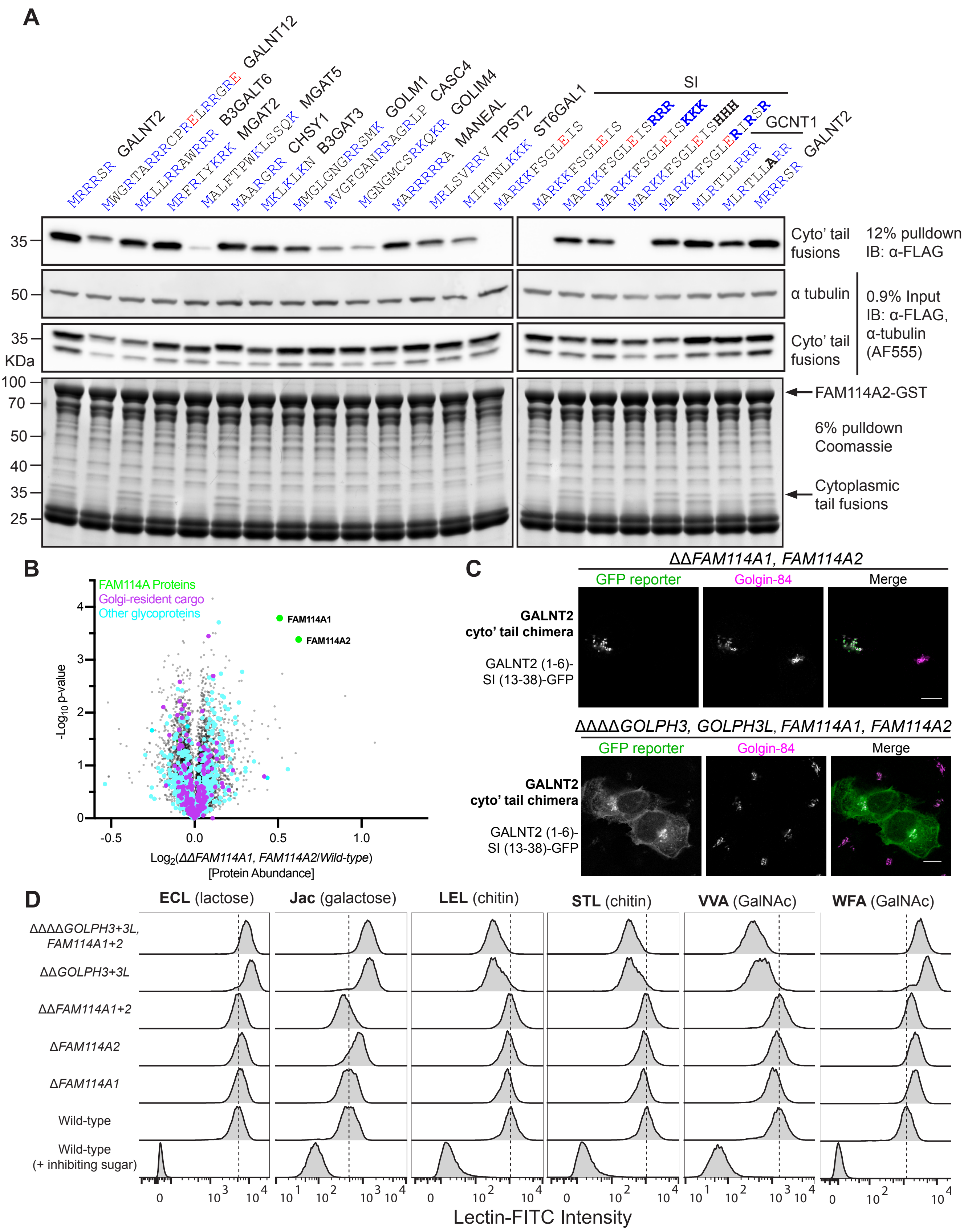

Journal of Cell Science • Supplementary information

Fig. S2. Analysis of cells lacking FAM114A1 and FAM114A2.

(A) Replicate of Fig. 2C showing binding studies to test the ability of FAM114A2-GST to pulldown different cytoplasmic tail chimeras from HEK293T cell lysate. Tail sequences and their corresponding gene names (above) are coloured in blue (positive) or red (negative) according to their predicted charge at a cytosolic pH of 7.4.

(B) Volcano plot comparing the relative abundance of individual proteins in *ΔΔFAM114A1, FAM114A2* U2OS cells vs the wild-type parental control cell line. The Z-score was normalized based on the median and a Student's t-test was applied to generate p-values. N=2 (full data in Table S1).

(C) Confocal micrographs showing a GFP-tagged cytoplasmic tail chimera expressed in *ΔΔFAM114A1, FAM114A2* and *ΔΔΔΔFAM114A1, FAM114A2, GOLPH3, GOLPH3L* U2OS cells. The GFP signal was enhanced with a GFP booster and golgin-84 was stained as a Golgi marker.

(D) Density curves generated from flow cytometry analysis of different CRISPR knockout U2OS cell lines subjected to cell surface stains with a panel of different FITC-conjugated lectins (see top, in bold). Specificity of the lectin was validated in which cells were stained in the presence of saturating concentrations of a competing sugar (see top, brackets). Density curves are normalised to the mode value for each treatment. Dotted lines mark the mode intensity value for wild-type cells. At least 10,000 events were collected for each cell line. Singlets were gated for based on forward and side scatter, dead cells were excluded using a fixable viability stain. Shown are the results of a single repeat of a triplicate.

Table S1. Mass spectrometry data as plotted in Figs. 1B, 1E and 1F; Figs. 4B-4F; and Fig. S2B.

Available for download at
https://journals.biologists.com/jcs/article-lookup/doi/10.1242/jcs.262160#supplementary-data

Table S2. Plasmids and antibodies used in this study.

Available for download at
https://journals.biologists.com/jcs/article-lookup/doi/10.1242/jcs.262160#supplementary-data
