# Supplementary material for: The FAM114A proteins are adaptors for the recycling of Golgi enzymes
Source: J Cell Sci. 2024 Sep 6;137(17):jcs262160. doi: 10.1242/jcs.262160 (PMC11441981; doi:10.1242/jcs.262160)
Supplement: Supplementary file 1 [file joces-137-262160_review_history.pdf]

Journal of Cell Science

The FAM114A proteins are adaptors for the recycling of Golgi enzymes

Lawrence G. Welch, Nadine Muschalik and Sean Munro
DOI: 10.1242/jcs.262160

Editor: Giampietro Schiavo

Review timeline
Original submission: 27 March 2024
Editorial decision: 13 May 2024
First revision received: 26 July 2024
Accepted: 1 August 2024

Original submission

First decision letter

MS ID#: JOCES/2024/262160

MS TITLE: The FAM114A proteins are adaptors for the recycling of Golgi enzymes

AUTHORS: Lawrence G Welch, Nadine Muschalik, and Sean Munro

We have now reached a decision on the above manuscript.

To see the reviewers' reports and a copy of this decision letter, please go to: https://submit-jcs.biologists.org and click on the 'Manuscripts with Decisions' queue in the Author Area. (Corresponding author only has access to reviews.)

As you will see, the reviewers gave favourable reports but raised some critical points that will require amendments to your manuscript. I hope that you will be able to carry these out because I would like to be able to accept your paper, depending on further comments from reviewers.

Please ensure that you clearly highlight all changes made in the revised manuscript. Please avoid using 'Tracked changes' in Word files as these are lost in PDF conversion.

I should be grateful if you would also provide a point-by-point response detailing how you have dealt with the points raised by the reviewers in the 'Response to Reviewers' box. Please attend to all of the reviewers' comments. If you do not agree with any of their criticisms or suggestions please explain clearly why this is so.

Reviewer 1

Advance summary and potential significance to field

This study reports the identification of two previously uncharacterized paralogous proteins, FAM114A1 and FAM114A2 that appear to serve as adaptors for the interaction of a subset of transmembrane Golgi proteins with the COPI vesicle coat. First identified in a BioID-based assay using the Golgi resident GMAP-210 as bait, the interaction of both proteins with multiple Golgi proteins was confirmed by affinity precipitation/mass spectroscopy. Although smaller, FAM114A2 binds a larger set of Golgi proteins than does FAM114A1. As previously reported for another Golgi

© 2024. Published by The Company of Biologists under the terms of the Creative Commons Attribution License (https://creativecommons.org/licenses/by/4.0/).

adaptor, GOLPH3, FAM114A2 appears to recognize a cluster of basic amino acids close to the transmembrane domain. Although the biochemical data are quite clean and impressive, CRISPR/Cas9 knockouts of either gene individually or both together had only a minor impact on protein glycosylation in two different human cell lines. However, knockout of the single Drosophila FAM114A ortholog CG9590 did reveal a significant decrease in glycosylation of the secreted mucin Sgs3 and a reduced size of secretory granules in the salivary gland. Taken together, the results suggest that mammalian FAM114As and their Drosophila ortholog are novel and bona fide COPI cargo adaptors that, based on immunostaining, function in the early secretory pathway.

The data as presented are clear and convincing and support the conclusions drawn by the authors. It is disappointing (as I'm sure it was for the authors) that there was so little effect of even combinatorial knockouts in human cells, but as the authors point out the function of these proteins may be more important in some cell types than others. By analogy with the Drosophila ortholog, is it possible that they are most important in cells with a regulated secretory pathway?

I have only a few criticisms of the study:

1. Both GOLPH3 and the FAM114A proteins appear to recognize basic patches in the juxtamembrane region of their putative cargo proteins. It would therefore be useful to directly compare the cargos recognized by FAM114A1 and A2 with those recognized by GOLPH3, in a volcano plot format. How different are they?
2. Similarly, the pulldown assays shown in Fig. 2 should be repeated with GOLPH3 and directly compared to FAM114A2.
3. Fig. 4 should include higher magnification (and better resolved) images of the relative distribution of FAM114A and the other Golgi markers shown.
4. The size distribution of the 3rd instar salivary glands (Fig. 6) should be quantified.

Comments for the author

Although loss of both FAM114A proteins has only minor effects on secretion, this reviewer believes that their discovery and initial characterization are still important contributions to the field.

<u>Reviewer 2</u>

Advance summary and potential significance to field

The authors characterize FAM114A protein as a Golgi protein that traffics in COPI vesicles and associates with a subset of Golgi resident proteins. The authors use their established Golgin chimera fused to the biotin ligase BirA targeted to mitochondria in HEK293 cells to select Golgi-derived vesicles and a variation of BioID with a biotin pulse and isolation of proteins with streptavidin and protein characterization by LC-MS/MS. The authors report n=2 biological replicates with spectral counts to estimate protein abundance. They select for further study FAM114A2. GST-tagged FAM114A1 and FAM114A2 pulldowns from lysates after expression in HEK293T cells with proteins characterized by LC-MS/MS. To sort through the candidate associated proteins GST-tagged FAM114A coated beads were tested for association with the tail of GALNT2 that was specific to FAM114A2 and with further specificity shown for FAM114A2 for tails of type II Golgi resident membrane proteins with polybasic residues in the tail. CRISPR/Cas9 deletion of FAM114A genes in U2OS cells followed by Multiplex MS for protein characterization as well as flow cytometry, IF, and lectin labelling studies also done after siRNA knockdown of FAM114A proteins in USO2 cells all with little effect concluded. The authors studied CG9590 protein in Drosophila as a FAM114A protein orthologue. GST-tagged CG9590 protein associations in S2 cell lysates characterized several proteins reported as Golgi membrane proteins as well as COPI coatomer protein subunits. Expression of GFP- CG9590 in the Drosophila salivary gland, and concluded to be localized to the cis-Golgi and required for O-linked glycosylation of Sgs3 glycoprotein with loss of CG9590 concluded to result in loss of Golgi labelling by VVA and altered PNA lectin staining. The authors conclude that FAM114A proteins that include CG9590 in Drosophila allow COPI vesicles to transport resident Golgi proteins to different parts of the Golgi.

Taken together the authors' work on Fam114A does add to the knowledge of Golgi function.

© 2024. Published by The Company of Biologists under the terms of the Creative Commons Attribution License (https://creativecommons.org/licenses/by/4.0/).

Comments for the author

For the MitoID experiments, could the authors check if the doxycyclin for 24 hours followed by another 9 hours with additional nocodazole causes a change in gene expression by any of the Ire1, Atf6, or PERK signalling pathways? For the protein characterization after SDS PAGE and Coomassie staining could the stained gels be shown for the various samples? For the spectral counts, variation seems high in Figure 1B and Table S1. Could the authors expand their studies to n=3 biological replicates with means +/- SD indicated?
Supplementary Table S1 could be considered for a table legend that may be needed for ease of navigation through the rows, columns, and tabs. For all the proteomics experiments, can the authors indicate if the raw data have been submitted to a public depository? What is the significance of the highlighted rows in the Figure 1 B tab of Table S1 since some of the proteins are not in Figure 1B in the text? Can the authors comment on proteins such as TFIID subunit 3 or nucleolar protein of 40kDa highlighted in Table 1, Figure 1 tab? Furthermore, Table S1 is not indicated in the text of the manuscript.
For the GST-tagged FAM114A1 and FAM114A2 pulldowns, can the Coomassie blue-stained gels be indicated?
For navigation ease in Table S1, Figure 1E and 1F tab could the author s indicate with highlights the proteins selected in the text Figures 1E, 1F? Can the authors comment on unexpected pulldown proteins such as Elongation factor 1 beta,1 gamma, ATP synthase subunits, mitochondrial nuclease, NUMA1, etc? Have the authors checked if the Methyltransferase 26 protein (C16orf13) is Golgi localized?

For Fig2C, can the authors extend the study to n=3 with quantification indicated and means+/-SD? Can the methodology for Fig2 be added to the Materials and Methods section? Are the primary data for Figure S2A missing from Table S1? Can the experiments be expanded to n=3 biological replicates?

For Figure 3A, can this be done for n=3 with quantification? For Figure 23 B can a supplementary figure show the n=3 experiments? For Figure 3C, n=3 replicates with quantification may be considered. For Fig3E, could the authors also indicate SD besides SEM?

For Figures 4 B, C, and D, can the authors highlight in Table S1 in the appropriate tab the selected proteins shown in the Figures? In Figure 4B is Prat that is indicated, the chloroplast import protein? Is CG3961 also indicated known or predicted by the authors to be Golgi localized or is this considered in the experiments of Figure 4 D?

For Figure 6A could n=3 experiments be indicated with quantification? For Figure 6B the authors indicate Golgi staining which is difficult to see in the submitted figures, Can higher magnifications be shown with n= 3 biological replicates and quantification?

The AlphaFold figures should be retained and add considerably to the understanding of predicted mechanisms.

<u>Reviewer 3</u>

Advance summary and potential significance to field

A central question about the Golgi apparatus is how subsets of resident proteins are captured into specific classes of retrograde COPI coated vesicles. COPI is believed to function together with cargo adaptors such as GOLPH3+3L. Here, the authors performed proximity biotinylation with the vesicle tethering portion of the golgin GMAP-210 to identify a previously uncharacterized cytosolic protein called FAM114A2. This protein and its paralogue FAM114A1 seem to be Rab2 effectors, and they bind to positively charged cytosolic tails of Golgi resident proteins. A knockout experiment indicated that the FAM114A proteins are not crucial for Golgi function in mammals. However, a Drosophila FAM114A orthologue binds to COPI and plays a role in glycosylation. Thus, FAM114A proteins are candidates for a novel class of COPI adaptors.

© 2024. Published by The Company of Biologists under the terms of the Creative Commons Attribution License (https://creativecommons.org/licenses/by/4.0/).

This study is carefully done and well presented. It is a useful contribution to the field. I have no suggestions for further experiments, but I do have some comments about the presentation as indicated below.

Comments for the author

The following minor comments are for the authors' consideration. I do not need to see the revisions.

1. Does the geometry make sense if FAM114A binds simultaneously to membrane-associated Rab2 and to a membrane-proximal cytosolic tail?

2. Some of the Golgi enzymes examined here, including GALNT2 and ST6GAL1, were previously described by the Munro lab as GOLPH3+3L clients. Is the idea that FAM114A and GOLPH3+3L recognize overlapping sets of proteins for intra-Golgi recycling? A less optimistic interpretation is that the binding assays do not reliably capture the physiological interactions. This issue should be addressed in the Discussion.

3. Figure 3E is visually misleading. The vertical axis should start at 0 rather than 0.5.

4. In Figure 5C, it is surprising that FAM114A seems to be earlier in the Golgi than GMAP. Doesn't this result argue against the idea that FAM114A captures Golgi proteins in retrograde COPI vesicles that bind to GMAP?

5. On page 9, I don't understand this phrase: "The major glue protein Sgs3 showed increased gel mobility in the FAM114A mutant and a deficiency removing the FAM114A gene".

6. On page 12, "to direct binding or proteins" should presumably read "to direct binding of proteins".

7. The authors state that they "conclude that the FAM114A proteins act as adaptors to help recruit Golgi enzymes into COPI-coated vesicles". This idea is attractive and is a reasonable interpretation of the data, but the evidence is still tentative. I would suggest "propose" instead of "conclude".

First revision

Author response to reviewers' comments

Response to Reviewers' Comments.

We are very grateful to the reviewers for their positive comments on our work and their constructive suggestions for improvements. We have followed their suggestions as described below.

Reviewer 1 Advance Summary and Potential Significance to Field:

The data as presented are clear and convincing and support the conclusions drawn by the authors. It is disappointing (as I'm sure it was for the authors) that there was so little effect of even combinatorial knockouts in human cells, but as the authors point out the function of these proteins may be more important in some cell types than others. By analogy with the Drosophila ortholog, is it possible that they are most important in cells with a regulated secretory pathway?
We agree that a role in regulated secretion is an interesting possibility, but we have no direct evidence to confirm this and moreover the two proteins are reported to be expressed in a wide range of tissues. We have added a brief note to the Discussion to note these points.

I have only a few criticisms of the study:

© 2024. Published by The Company of Biologists under the terms of the Creative Commons Attribution License (https://creativecommons.org/licenses/by/4.0/).

1. Both GOLPH3 and the FAM114A proteins appear to recognize basic patches in the juxtamembrane region of their putative cargo proteins. It would therefore be useful to directly compare the cargos recognized by FAM114A1 and A2 with those recognized by GOLPH3, in a volcano plot format. How different are they?

This was most easily done for the proteins from *Drosophila* as the affinity chromatography experiments were done in parallel at a comparable scale. We have thus included two new graphs that compare the proteins enriched with GOLPH3 to those enriched by FAM114A. These show that there is little strong preference apart from COPI binding to FAM114A (as is already noted), and a few Golgi enzymes apparently preferring GOLPH3. Overall, the proteins that are strongly enriched by both proteins are all Golgi resident membrane proteins. These graphs are shown in Figure 4E, and we have added text to the relevant part of the results to discuss them

2. Similarly, the pulldown assays shown in Fig. 2 should be repeated with GOLPH3 and directly compared to FAM114A2.

Pulldown assays testing GOLPH3 with 17 of the 20 cytoplasmic tails shown in Fig. 2 were reported in our earlier paper on GOLPH3 (Welch et al. (2021) JCB, 220:e202106115 - available as open access). We apologise for not making this clear in the text and for not comparing the results with the two proteins. We have now added text to the relevant part of the Results to mention the previous work and to note that the overall pattern of binding is similar, and that where there are differences it is that some (but not all) protein tails bind better to GOLPH3.

3. Fig. 4 should include higher magnification (and better resolved) images of the relative distribution of FAM114A and the other Golgi markers shown.

We have replaced all of the images comparing FAM114A and Golgi markers with new ones obtained using an Airyscan confocal that gives a higher resolution than the conventional confocal used for the previous versions (note that it is Fig.5C rather than Fig.4). We have quantified these images, and also show higher magnification inserts. The markers are now much better resolved and so this was an excellent suggestion.

4. The size distribution of the 3rd instar salivary glands (Fig. 6) should be quantified.

We have added to Figure 6 a quantitation of the effect of the FAM114A mutation on the size of the secretory granules in the salivary gland. This shows a statistically significant reduction in granule size in the mutant which is rescued by expression of FAM114A. This is consistent with a reduction in granule size reported previously for other mutants with defects in glycosylation of the glue proteins that are stored in the granules.

Reviewer 2 Advance Summary and Potential Significance to Field:

Taken together the authors' work on Fam114A does add to the knowledge of Golgi function.

Reviewer 2 Comments for the Author:

For the MitoID experiments, could the authors check if the doxycyclin for 24 hours followed by another 9 hours with additional nocodazole causes a change in gene expression by any of the Ire1, Atf6, or PERK signalling pathways?

Doxycyclin and nocodazole are both widely used in cell biological experiments, and even if they were to induce ER stress it is not clear why this should affect the ability of golgins to capture vesicles. We and others have previously reported that such capture can be seen in cells where we did not use doxycyclin to induce gene expression, and also that some capture occurs even without the use of nocodazole to bring the Golgi closer to more mitochondria (Wong and Munro, 2014; Sato et al 2015). The localisation of FAM114A proteins to the Golgi has been previously reported by ourselves and others, and their contribution to Golgi organisation is tested in this paper, and none of these experiments involve either doxcyclin

© 2024. Published by The Company of Biologists under the terms of the Creative Commons Attribution License (https://creativecommons.org/licenses/by/4.0/).

or nocodazole. Finally, in our previous work, the MitoID approach has correctly identified numerous Rab effectors that have been confirmed by ourselves and others. Nonetheless, we agree that, like any cellular treatment, some transcriptional responses may well occur (doxycycline is actually believed to affect mitochondria rather than the ER as it is an antibiotic, and nocodazole inevitably perturbs the cell cycle). We have thus added a sentence to note that for the MitoID experiment the cells may be experiencing some stress due the reagents used, but these reagents are not included in subsequent experiments.

For the protein characterization after SDS PAGE and Coomassie staining could the stained gels be shown for the various samples? For the spectral counts, variation seems high in Figure 1B and Table S1. Could the authors expand their studies to n=3 biological replicates with means +/- SD indicated?

The Coomassie-stained gels were not recorded prior to mass-spectrometry as the latter method measures the same information (protein size and abundance), with added advantage of identifying the protein as well. Spectral counts tend to be variable between biological replicates as mass-spectrometry is an inherently stochastic method. There is inevitably some variation in the precise amounts of the trace non- specific binding shown by very abundant cellular proteins (the “CRAPome”), and for any protein the fact that the spectrometer does not analyse the passing peptides whilst one is being identified means that there is an element of chance as to how many times a peptide is fragmented for analysis. We are not seeking statistical confidence for the observation of FAM114A amongst the hits as its role in the Golgi is instead directly validated the experiments that follow in the rest paper.

Supplementary Table S1 could be considered for a table legend that may be needed for ease of navigation through the rows, columns, and tabs. For all the proteomics experiments, can the authors indicate if the raw data have been submitted to a public depository?

This is an excellent suggestion, and an overview has been added that explains the different tabs, and we have also added highlighting to several of the tables following the reviewer’s helpful suggestions. As for the raw data, this has been submitted to Pride and the relevant accession number is provided in the revised paper.

What is the significance of the highlighted rows in the Figure 1 B tab of Table S1 since some of the proteins are not in Figure 1B in the text?

Figure 1B shows the fifteen strongest hits, with the complete table being in Table S1. In both cases the highlighting indicates proteins reproducibly labelled with GMAP but absent from the negative control (wild type WT)) control and substantially reduced (ie 0 or 1 spectral count) by the W4A mutation in the GMAP-210 vesicle capture motif. This is stated in the legend to Figure 1B and in box on the corresponding tab in Table S1.

Can the authors comment on proteins such as TFIID subunit 3 or nucleolar protein of 40kDa highlighted in Table 1, Figure 1 tab?

These are cytosolic proteins of the sort which can appear as background in mass-spectroscopic analysis of material isolated from whole cell lysates. Presumably by chance these two proteins gave more spectra with GMAP-210. A comment to this effect has been added to the Figure Legend.

Furthermore, Table S1 is not indicated in the text of the manuscript.

We apologise for this oversight, and the table is now referred to in the context of each of the relevant figures.

For the GST-tagged FAM114A1 and FAM114A2 pulldowns, can the Coomassie blue- stained gels be indicated?

As explained above, we did not record the Coomassie-stained gels prior to mass spectrometry, especially as the gels were only run a short distance to reduce the size of slices prior to trypsin

© 2024. Published by The Company of Biologists under the terms of the Creative Commons Attribution License (https://creativecommons.org/licenses/by/4.0/).

digestion and so little is clearly visible on the gel apart from the location of the lanes.

For navigation ease in Table S1, Figure 1E and 1F tab could the authors indicate with highlights the proteins selected in the text Figures 1E, 1F?

We have added highlighting to the Figure 1E and 1F tab of Table S1 to indicate the proteins named in panels E and F in Figure 1.

Can the authors comment on unexpected pulldown proteins such as Elongation factor 1 beta,1 gamma, ATP synthase subunits, mitochondrial nuclease, NUMA1, etc? Have the authors checked if the Methyltransferase 26 protein (C16orf13) is Golgi localized?

Proteins obtained from whole cell lysates by affinity chromatography or immunoprecipitation always include trace amounts of very abundant proteins such as translation factors, abundant enzymes, cytoskeletal proteins etc. This is referred to as “background binding”, or “the CRAPome”. The precise amount of each protein brought down can vary depending on washing conditions or factors that affect non-specific binding such as the charge or “stickiness” of the bait. Thus, it is typical to see some very abundant proteins that appear enriched for such reasons. These are clearly distinct from specific binders such as the Golgi enzymes found amongst them as these are present in the starting lysate at much lower levels (typically 50-100 fold), and so their presence reflects a much great degree of enrichment.

We have not studied C16orf13 further, and published organelle proteomics experiments report it to be cytosolic in contrast to the FAM114A proteins which are found to be Golgi associated. The fact that knockout mice are viable and fertile further lowered our enthusiasm. We now state these facts in the Results section.

For Fig2C, can the authors extend the study to n=3 with quantification indicated and means+/-SD? Can the methodology for Fig2 be added to the Materials and Methods section? Are the primary data for Figure S2A missing from Table S1? Can the experiments be expanded to n=3 biological replicates?

The experiment is intended to give a qualitative rather than quantitative comparison of interactions that are being tested in vitro and hence may not be at precisely the same conditions as in the cell. In accordance with standard practice at J. Cell Science and indeed other reputable cell biological journals, we now indicate for this experiment how many replicates it is representative of. In addition, to reassure readers of our ability to pipette samples and run gels reproducibly, we show a replicate of the experiment in supplemental Figure S2A.

The methodology for Figure 2 was published in a previous paper that is cited and available online through open access. The data shown in Figure S2A (now Figure S2B) has been added as a Tab in Table S1, and the raw data submitted to the Pride Repository. We did two replicates and the results were not encouraging enough to expend further resources to cover another round of TMT labelling and mass- spectrometry.

For Figure 3A, can this be done for n=3 with quantification?

The figure shows validation of CRISPR knockouts. Given that the proteins we intended to target are clearly absent following CRISPR-Cas9, then the correct genes have been targeted and quantification of absence seems of little value. In accordance with standard practice, we now state in the figure legend that the gel is representative of three replicates, and that the clones were validated by PCR and sequencing.

For Figure 3B can a supplementary figure show the n=3 experiments?

The result shown is essentially a negative one (ie that deletion of FAM114A1 and FAM114A2 does not affect the retention of a protein with the tail of GALNT2), and we can reassure the reviewer that we did not deliberately hide a replicate that showed something more interesting! As with the other experiments we state in the figure legend the number of

© 2024. Published by The Company of Biologists under the terms of the Creative Commons Attribution License (https://creativecommons.org/licenses/by/4.0/).

replicates of which the data shown is representative.

For Figure 3C, n=3 replicates with quantification may be considered.

The purpose of this blot is to show that both proteins are expressed in both cells but at a different ratio. This is a qualitative rather than quantitative observation, and the ratio of two proteins in the same sample would not in any case be affected by slight variations in loading and so we feel that further replicates and quantitation would not add anything. We also have added to the discussion a brief note to the effect that the ratio of the two proteins appears to vary somewhat between cell types and tissues.

For Fig3E, could the authors also indicate SD besides SEM?

Figure 3E now shows SD and the Figure Legend has been adjusted accordingly.

For Figures 4 B, C, and D, can the authors highlight in Table S1 in the appropriate tab the selected proteins shown in the Figures?

This is an excellent suggestion and we have now added highlights to Table S1 to label the selected proteins shown in the panels in Figure 4.

In Figure 4B is Prat that is indicated, the chloroplast import protein? Is CG3961 also indicated known or predicted by the authors to be Golgi localized or is this considered in the experiments of Figure 4 D?

We apologise for not making it clear that *Drosophila*, the organism examined in this part of the paper is an animal rather than a plant and so does not have chloroplasts. We have now added a note to the legend for Figure 4, to explain that Prat is a glutamine amidotransferase from the purine biosynthesis pathway. We highlighted it as it is the one protein apart from known components of membrane traffic that was highly enriched by affinity chromatography with Drosophila FAM114A. Its significance is unclear and it may well represent spurious binding, potentially to the COPI that binds to FAM114A, but we thought that we should note it. CG3961 is ER localised and we apologise for erroneously including it. We have reviewed all the indicated proteins to ensure that they are all Golgi residents or plausibly recycling between ER and Golgi.

Three CG numbers remain, and their identity is provided in the figure legend.

For Figure 6A could n=3 experiments be indicated with quantification? For Figure 6B the authors indicate Golgi staining which is difficult to see in the submitted figures, Can higher magnifications be shown with n= 3 biological replicates and quantification?

For Figure 6A we now state in the figure legend that the image shown is representative of four repeats. For the staining of the Golgi by the lectin VVA that was shown in Figure 6B we now show new images double labelled with a Golgi marker. This shows that the punctate distribution reflecting labelling of the Golgi stack is lost in the FAM114A mutant and restored by expression of a rescue construct (now Figure 6D). We also show higher magnification insets to make clear the close proximity of the VVA staining to that of the Golgi marker GM130.

The AlphaFold figures should be retained and add considerably to the understanding of predicted mechanisms.

The figures have been retained.

Reviewer 3 Advance Summary and Potential Significance to Field:
A central question about the Golgi apparatus is how subsets of resident proteins are captured into specific classes of retrograde COPI coated vesicles. COPI is believed to function together with cargo adaptors such as GOLPH3+3L. Here, the authors performed proximity biotinylation with the vesicle tethering portion of the golgin GMAP-210 to identify a previously uncharacterized cytosolic protein called FAM114A2. This protein and its paralogue FAM114A1

© 2024. Published by The Company of Biologists under the terms of the Creative Commons Attribution License (https://creativecommons.org/licenses/by/4.0/).

seem to be Rab2 effectors, and they bind to positively charged cytosolic tails of Golgi resident proteins. A knockout experiment indicated that the FAM114A proteins are not crucial for Golgi function in mammals. However, a Drosophila FAM114A orthologue binds to COPI and plays a role in glycosylation. Thus, FAM114A proteins are candidates for a novel class of COPI adaptors.

This study is carefully done and well presented. It is a useful contribution to the field. I have no suggestions for further experiments, but I do have some comments about the presentation as indicated below.

Reviewer 3 Comments for the Author:

The following minor comments are for the authors' consideration. I do not need to see the revisions.
1. Does the geometry make sense if FAM114A binds simultaneously to membrane-associated Rab2 and to a membrane-proximal cytosolic tail?

We apologise for not discussing this properly. The predicted Rab2 binding side is away from the acidic part of the FAM114A surface and so would not interfere with binding to a short and basic cytosolic tail, and the unstructured hypervariable region of the Rab is long enough that the lipidated C-terminus of the Rab could remain in the membrane.
We have added a note to explain this and have also labelled the acidic faces in the panel in Fig S1 that shows the predicted structure of the Rab2:FAM114A complex.

2. Some of the Golgi enzymes examined here, including GALNT2 and ST6GAL1, were previously described by the Munro lab as GOLPH3+3L clients. Is the idea that FAM114A and GOLPH3+3L recognize overlapping sets of proteins for intra-Golgi recycling? A less optimistic interpretation is that the binding assays do not reliably capture the physiological interactions. This issue should be addressed in the Discussion.

Yes, our idea is that FAM114A and GOLPH3/3L recognise overlapping sets of enzymes. The in vitro binding data suggest that GOLPH3 has a somewhat broader range of interactors, as is now noted in the text (see response above to comment 2 from Reviewer 1). It is possible that there are FAM114A-specific clients that are found in specific tissues that remain to be identified. We are quite confident that the in vitro binding is not non-specific as the proteins most enriched by both proteins are all from the Golgi (see response above to comment 1 from Reviewer 1). Nonetheless, we agree that the in vitro binding assay may not capture all of the interactions made in vivo, and we have added a comment about this in the part of the Results that covers the comparison between FAM114A and GOLPH3 interactors.

3. Figure 3E is visually misleading. The vertical axis should start at 0 rather than 0.5.
We agree, and the axis has now been corrected.

4. In Figure 5C, it is surprising that FAM114A seems to be earlier in the Golgi than GMAP. Doesn't this result argue against the idea that FAM114A captures Golgi proteins in retrograde COPI vesicles that bind to GMAP?

Although FAM114A and GMAP do not have identical distributions the Golgi stack there is considerable overlap. It is possible that the FAM114A that is earlier than GMAP is in vesicles that have yet to be captured, or are heading back to the ER, or has been recruited by Rab2 prior to incorporation into vesicles as the stack matures. The organisation of the ER/Golgi region and of COPI vesicle biogenesis is clearly complex and remains only poorly understood and so there remains a lot of room to uncertainty and speculation. We have added a brief comment to the Discussion to cover this caveat.

5. On page 9, I don't understand this phrase: "The major glue protein Sgs3 showed increased gel mobility in the FAM114A mutant and a deficiency removing the FAM114A gene".

We apologise for the unclear wording. This has now been rephrased as "*Salivary gland*

© 2024. Published by The Company of Biologists under the terms of the Creative Commons Attribution License (https://creativecommons.org/licenses/by/4.0/).

proteins were prepared from flies lacking FAM114A and when separated by gel electrophoresis the major glue protein Sgs3 migrated faster than in wild type..".

6. On page 12, "to direct binding or proteins" should presumably read "to direct binding of proteins".

Corrected.

7. The authors state that they "conclude that the FAM114A proteins act as adaptors to help recruit Golgi enzymes into COPI-coated vesicles". This idea is attractive and is a reasonable interpretation of the data, but the evidence is still tentative. I would suggest "propose" instead of "conclude".

We agree that this wording is more appropriate and have made the relevant change.

Second decision letter

MS ID#: JOCES/2024/262160

MS TITLE: The FAM114A proteins are adaptors for the recycling of Golgi enzymes

AUTHORS: Lawrence G Welch, Nadine Muschalik, and Sean Munro

I am happy to tell you that your revised manuscript has been accepted for publication in Journal of Cell Science, pending standard publication integrity checks. Thank you for following reviewers' suggestion and for explaining the changes in your response.

© 2024. Published by The Company of Biologists under the terms of the Creative Commons Attribution License (https://creativecommons.org/licenses/by/4.0/).
